# Efficacy of rigosertib, a small molecular RAS signaling disrupter for the treatment of *KRAS*-mutant colorectal cancer

**DOI:** 10.20892/j.issn.2095-3941.2020.0532

**Published:** 2021-08-04

**Authors:** Xinyi Zhou, Qian Xiao, Dongliang Fu, Haochen Zhang, Yang Tang, Jinjie He, Yeting Hu, Xiangxing Kong, Fei Teng, Xiangrui Liu, Ying Yuan, Kefeng Ding

**Affiliations:** 1 Department of Colorectal Surgery and Oncology, Key Laboratory of Cancer Prevention and Intervention, Ministry of Education, the Second Affiliated Hospital of Zhejiang University School of Medicine, Hangzhou 310009, China; 2 Department of Medical Oncology, Key Laboratory of Cancer Prevention and Intervention, Ministry of Education, the Second Affiliated Hospital of Zhejiang University School of Medicine, Hangzhou 310009, China; 3 Hangzhou Oncocare Co Ltd, Hangzhou 310009, China; 4 Department of Pharmacology, Zhejiang University School of Medicine, Hangzhou 310058, China; 5 Cancer Center, Zhejiang University, Hangzhou 310009, China

**Keywords:** Colorectal cancer, *KRAS* mutation, rigosertib, therapeutic effect, RAS signaling

## Abstract

**Objective:**

Mutant KRAS, the principal isoform of RAS, plays a pivotal role in the oncogenesis of colorectal cancer by constitutively activating the RAF/MEK/ERK and PI3K/AKT pathways. Effective targeted therapies are urgently needed. We investigated whether rigosertib, a benzyl styryl sulfone RAS signaling disruptor, could selectively kill *KRAS*-mutant colorectal cancer cells.

**Methods:**

CCK-8 was used to determine the cell viability. Patient-derived tumor and cancer cell xenograft models were used to detect the inhibitory efficacy of rigosertib. Flow cytometry was used to evaluate the apoptosis and cell cycle progression. Apoptosis and cell cycle arrest markers were detected by Western blot. DCFH-DA was used to determine the reactive oxygen species. Immunohistochemistry staining and Western blot were performed to characterize RAS signaling markers in colorectal cancer tissues and cells.

**Results:**

Rigosertib (RGS) exhibited a cytotoxic effect against colorectal cancer cells, which was greater in *KRAS*-mutant cells. Furthermore, RGS induced mitotic arrest and oxidative stress-dependent apoptosis in *KRAS*-mutant DLD1 and HCT116 cells. Besides, RGS disrupted RAS signaling, and the inhibition of RAS/MEK/ERK was independent of cellular oxidative stress. Using patient-derived xenograft models, the response and tumor inhibition of RGS were significantly higher in the *KRAS*-mutant subgroup, while p-MEK, p-ERK, and p-AKT levels of RGS-treated tumors were significantly decreased. Finally, in a *KRAS*-mutant, chemotherapy-resistant patient-derived xenograft model, RGS showed a stronger therapeutic effect than the combination standard therapy involving fluoropyrimidine + oxaliplatin/irinotecan + bevacizumab.

**Conclusions:**

These data showed that targeting RAS signaling using RGS could be a therapeutic treatment for *KRAS*-mutant colorectal cancer patients.

## Introduction

Colorectal cancer (CRC) is the fifth most common cause of cancer-related mortality in China^1^. The prognosis for metastatic CRC (mCRC) is poor^2^, but the introduction of anti-epidermal growth factor receptor (EGFR) monoclonal antibodies to treat mCRC has significantly improved patient survival. However, clinical trials have shown that anti-EGFR monoclonal antibodies do not benefit *KRAS*-mutant mCRC patients^3^.

*KRAS*, one of the most frequently mutated oncogenes in CRC, is the main component of 3 members of the *RAS* family (*KRAS, NRAS*, and *HRAS*). This family encodes 4 highly homologous RAS isoforms: KRAS4A, KARS4B, NRAS, and HRAS (KRAS4A and KARS4B are splice variants of the *KRAS* gene)^4^. As a small GTPase, the RAS protein is stimulated by a receptor tyrosine kinase *via* guanine nucleotide exchange factors, and then the activated RAS stimulates downstream pathways; this process is highly regulated, and physiological feedback loops limit the duration of RAS activation. When mutated, the RAS protein is maintained in a constitutively active GTP-bound state, driving the downstream RAF/MEK/ERK (MAPK) kinase cascade and PI3K/AKT axis^5^, and enhancing cancer cell proliferation and survival. However, because of the lack of druggable cavities on the mutant RAS surface, the development of mutant RAS inhibitors is progressing slowly^6^. Other studies attempted to inhibit RAS/MEK/ERK and PI3K/AKT signals using a combination treatment of specific inhibitors; however, these trials failed because the combination treatment produced unacceptable toxicity^7^.

Rigosertib (RGS) is a non-ATP competitive multiple kinase inhibitor that suppresses proliferation of various tumor cells *in vivo* and *in vitro*^8^. The direct target of RGS is still unknown. Dai et al.^9^ reported that RGS inhibited diffuse large B cell lymphoma growth by cytoplasmic sequestration of sumoylated C-MYB/TRAF6 proteins, while Oussenko et al.^10^ correlated hyperphosphorylation of RanGAP1 with RGS-induced cell death. Recently, 2 different groups described novel mechanisms for RGS modulation of the RAS signaling pathway. Athuluri-Divakar et al.^11^ suggested that RGS, functioning as a RAS mimetic, blocked RAS/effector interaction, and directly inhibited RAS/MEK/ERK and RAS/PI3K/AKT signaling, while Ritt et al.^12^ reported that RGS induced oxidative-dependent, JNK-mediated indirect inhibition of the RAS/MEK/ERK pathway. Despite differences in the details, both theories support an important role of RGS in disrupting RAS signaling^13^.

The undruggable mutant *RAS* genes have been reported as key driver genes that induce apoptotic elimination, drive invasion, and maintain metastasis in CRC^14^; targeting the activated *RAS* in CRC by homologous recombination could significantly damage cancer cell proliferation and transforming capacity both *in vivo* and *in vitro*^15,16^. We therefore characterized the potential RAS-disrupting effect of RGS in *RAS*-mutant CRC. Because of the predominant mutation frequency of *KRAS* among all three *RAS* genes^4^ and the association of *KRAS* mutations with a higher risk of distant metastasis in CRC^17^, this study mainly focused on *KRAS*-mutant CRC. In this study, we determined whether RGS inhibited RAS signaling and selectively killed *KRAS*-mutant CRC cells.

## Materials and methods

### Chemicals and reagents

RGS was purchased from Selleck Chemicals (Houston, TX, USA). Etoposide and paclitaxel were purchased from Beyotime Biotechnology (Shanghai, China). These chemicals were dissolved in dimethyl sulfoxide (DMSO; Sigma-Aldrich, St. Louis, MO, USA) and stored at -20 °C.

### Cell culture

Human CRC cell lines (SW48, Caco-2, DLD1, HCT116, LOVO, SW620, and SW480) were obtained from the American Type Culture Collection (Rockville, MD, USA) during October 2016. Following receipt, the cells were grown and frozen as seed stocks. The cells were passaged for a maximum of 3 months, after which new seed stocks were thawed. Cell lines were authenticated using DNA fingerprinting (using a variable number of tandem repeats). Caco-2 cells were cultured in a minimal essential medium (Gibco, Carlsbad, CA, USA) with 20% fetal bovine serum (FBS) (Life Technologies, Carlsbad, CA, USA), and the other cell lines were maintained in RPMI 1640 medium (Gibco), supplemented with 10% FBS, 100 units/mL of penicillin and 100 mg/mL of streptomycin at 37 °C in a humidified atmosphere of 5% CO_2_. All cell lines were routinely screened for the presence of mycoplasma (Mycoplasma Detection Kit, Sigma-Aldrich).

### Cell viability analysis

Cell viability was analyzed using the Cell Counting Kit-8 (CCK-8) assay (Dojindo Laboratories, Tokyo, Japan). The cells were seeded into 96-well plates at a density of 5–10 × 10^3^ cells/well overnight. The working solution of RGS was diluted with complete medium with a maximal concentration of 0.1% DMSO. The cells were treated with the indicated concentrations of RGS for 48 or 96 h. After incubation, CCK-8 was added to each well, and the absorbance was measured using a microplate reader at 450 nm after incubation for an additional 2 h. Three replicate wells were measured for each group.

### Cell colony formation

Approximately 1,000 cells were seeded in a 6-well plate and incubated overnight. The cells were cultured with the medium changed every 96 h in the presence or absence of 50 nM RGS. After 2 weeks, the remaining colonies were analyzed after fixation and Crystal Violet staining.

### Lentiviral transduction and generation of stable cell lines

Lentivirus was produced by transfecting HEK293T cells with the psPAX2 packaging plasmid (Addgene plasmid #12260), pMD2.G envelope plasmid (Addgene plasmid#12259), and different mutant types of *KRAS* transfer plasmids including *KRAS-G12D*, *KRAS-G12V*, and *KRAS-G13D*, using pLVX-IRES-puro (#VT1464; YouBio, Xian, China) as the control. The cell supernatants were collected at 24 and 48 h after transfection and were used for infection or stored at -80 °C. To obtain stable cell lines, the cells were infected at 70%–80% confluence for 24 h with lentivirus diluted 1:1 with a normal cell culture medium in 96-well plates. After 24 h of infection, the supernatants were replaced with normal cell culture medium. After 48 h, the cells were transferred under puromycin selection for approximately 1 week in 24-well plates and passaged before use. Puromycin was used at 2 μg/mL to maintain the SW48 and Caco-2 cell lines.

### Assessment of cell cycle progression by flow cytometry

DLD1 and HCT116 cells were synchronized at the G1/S boundary by serum starvation for 48 h. After the indicated RGS treatments, the cells were collected by trypsinization, washed with phosphate-buffered saline (PBS), fixed in 70% ethanol, incubated with propidium iodine for 30 min, and analyzed using flow cytometry (FACS Canto II; BD Biosciences, San Jose, CA, USA).

### Mitochondrial fractionation and analysis

Mitochondria-enriched fractionation was performed using a Mitochondria Isolation Kit (Thermo Fisher Scientific, Waltham, MA, USA) in accordance with the manufacturer’s instructions. The fractions of RGS-treated DLD1 and HCT116 cells were examined by Western blot using anti-Bax, anti-Cyt *c*, anti-cytochrome c oxidase (COX) IV, and anti-glyceraldehyde 3-phosphate dehydrogenase (GAPDH) antibodies.

### Measurement of reactive oxygen species (ROS) in cells and mitochondria

To assess the production of cellular and mitochondrial ROS, DLD1 cells were seeded on 6-well culture plates with coverslips at a density of 2 × 10^5^ cells, grown overnight, and then treated with RGS for the indicated times. Following incubation, the cells were incubated with DCFH-DA and Mito-Tracker for 1 h, and drops of anti-fade mounting medium were applied to the coverslips. Cellular images were captured by fluorescence or confocal microscopy (LSM 510; Carl Zeiss, Oberkochen, Germany).

### Measurement of apoptosis using the annexin V-propidium iodide assay

Measurement of cell apoptosis used annexin V, using the FITC Apoptosis Detection Kit (Dojindo Laboratories, Kumamoto, Japan), in accordance with the manufacturer’s instructions. Briefly, after RGS treatment, the cells were harvested, washed, and then resuspended in a binding solution (containing 5 μL of annexin V-FITC and 5 μL of propidium iodide), followed by incubation at room temperature in the dark for 15 min. Analyses were conducted within 1 h using a flow cytometer (FACS Canto II; BD Biosciences).

### Ethics approval and consent to participate

Written informed consent was obtained from all patients, and the study was approved by the Ethics Committee of the Second Affiliated Hospital of Zhejiang University School of Medicine (Approval No. 2020.609).

### Establishment of a bank of patient-derived xenograft models

Fresh surgical specimens (P0 = passage zero) were obtained from the operating room, and implanted subcutaneously into the flanks of 5–6-week-old female nude mice. Once the subcutaneous tumors (P1) grew to 500 mm^3^, the tumor fragments were harvested and replanted into other mice for passage (from P1 to P2), and the remaining tumor specimens were cryopreserved in a refrigerator. Tumor tissues from generation P1 or P2 were used to evaluate drug efficacy.

### Mouse xenograft colorectal cancer model

All animal procedures were performed in accordance with protocols reviewed and approved by the Animal Ethics Committee of the Second Affiliated Hospital of Zhejiang University School of Medicine (Approval No. 2020.035). Five- to 6-week-old female nude mice were purchased from SLAC Laboratory Animal Company (Shanghai, China). SW48 and DLD1 cells (1 × 10^6^) were suspended in PBS and injected subcutaneously into the mice. Tumor growth was monitored daily until the tumor was palpable (50–100 mm^3^). The mice were then randomized into 2 groups, and each group received PBS or RGS (100 mg/kg) by intraperitoneal injection. Body weight and tumor size were measured every 3 days. Once the tumor size reached 15–20 mm in any dimension, or the animals became ill, tumor fragments were harvested. Tumor volumes were calculated using the following formula: tumor volume (mm^3^) = L × S × S/2, where L is the long axes of the tumor and S represents the short axes of the tumor. After 4 weeks of administration, mice were sacrificed, and the tumors were excised, photographed, and further fixed in 10% neutral formalin and embedded in paraffin.

### Immunohistochemistry staining

Immunohistochemistry (IHC) was performed as we previously described^18^ using the corresponding primary antibodies. The results of IHC staining were reviewed and scored by 2 independent pathologists who were blinded to the study. The IHC staining level was evaluated using the immunoreactive score (IRS)^19^, which was calculated in a double grading system involving the staining intensity and percentage of positively stained cells. IHC staining intensity was scored from 0 to 3 (0 = negative, 1 = weak, 2 = moderate, and 3 = strong). The percentage of stained cells was graded as 1 when 0%–25% of the cells were stained, 2 when 26%–50% of cells were stained, 3 when 51%–75% of cells were stained, and 4 when 76%–100% of the cells were stained. Multiplying both parameters resulted in the IRS.

### Western blot

Total protein was extracted from CRC cells or PDX tissues after RGS treatments, and protein concentrations were determined using the Pierce BCA Protein Assay Kit (Thermo Fisher Scientific). Protein samples were subjected to 10%–12% SDS-PAGE and transferred to a polyvinylidene fluoride membrane (Bio-Rad, Hercules, CA, USA). After blocking with 5% nonfat milk for 1 h at room temperature, the membranes were incubated with appropriate primary antibodies overnight at 4 °C. Primary antibodies against cleaved caspase 3, cleaved caspase 9, PARP, p-ERK1/2 (T202/204), p-AKT (S473), ERK1/2, and AKT were purchased from Cell Signaling Technology (CST; Danvers, MA, USA) (all dilutions: 1:1,000). Anti-Bax, anti-Cyt *c*, and anti-COX IV (all dilutions, 1:1,000) were purchased from Abcam (Cambridge, MA, USA). Primary antibodies against p-MEK1 (S217/221), MEK1/2, cyclin B1, p-CDK1 (Y15), CDK1, p-CHK1 (S296), p-CHK2 (T86), and p-Histone H3 (S10) (all dilutions, 1:1,000) were purchased from Beyotime Biotechnology. A mouse anti-GAPDH monoclonal antibody (CST; dilution: 1:1,000) was used as the loading control. Following incubation with a secondary antibody conjugated to horseradish peroxidase for 1 h at room temperature, the immunoreactive bands were visualized using the enhanced chemiluminescence detection system (Thermo Fisher Scientific).

### Statistical analysis

All data were presented as a mean ± standard error of the mean of 3 independent experiments. Statistical analysis was performed using Student’s *t*-test or analysis of variance with multiple comparisons using Prism, version 6.0 software (GraphPad, La Jolla, CA, USA). The differences were considered significant at *P* < 0.05, *P* < 0.01, and *P* < 0.001.

### Data availability

The data sets used for the current study are available from the corresponding author upon reasonable request.

## Results

### Differential sensitivity to RGS of RAS wild-type and KRAS-mutant colorectal cancer cell lines *in vitro* and *in vivo*

**Figure 1:**
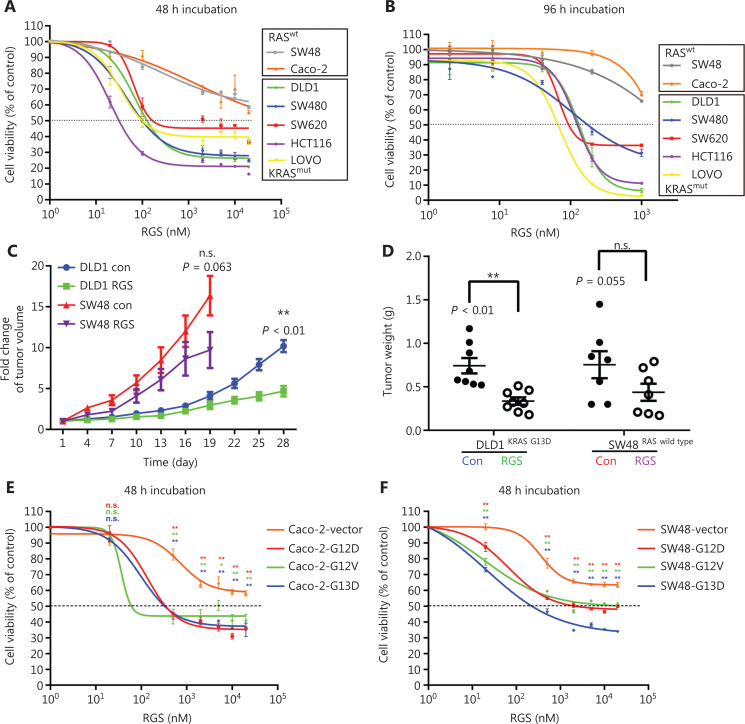
The anti-tumor effect of rigosertib (RGS) in a panel of human colorectal cancer (CRC) cell lines *in vitro* and *in vivo*. The cell viabilities of 2 *RAS* wild-type CRC cell lines (Caco-2 and SW48) and 5 *KRAS*-mutant cell lines (DLD1 KRAS^G13D^, SW480 KRAS^G12V^, SW620 KRAS^G12V^, HCT116 KRAS^G13D^, and LOVO KRAS^G13D^) treated with different concentrations of RGS for 48 h (A) and 96 h (B) were assessed. The anti-tumor effects of RGS in DLD1 and SW48 xenograft mouse models were assessed by measuring tumor volumes every 3 days after palpable tumors reached 50–100 mm^3^. Tumor growth was significantly inhibited in the RGS-treated DLD1 xenograft mice (*N* = 8) compared to the RGS-treated SW48 xenograft mice (*N* = 7). Because of the overgrowth of SW48 xenograft tumors, which almost reached the maximum size (15–20 mm in any dimension), we ended the experiment on day 19 in consideration of the ethical treatment of the animals (C). Tumor weight decreased in mice treated with RGS in the DLD1 xenograft model (0.336 ± 0.111 g *vs* 0.743 ± 0.233 g) but not in the SW48 xenograft model (D). His-tagged KRAS G12D-, G12V-, and G13D-mutant oncoproteins were stably expressed in Caco-2 (E) and SW48 cells (F), and the viabilities of mutant *KRAS*-expressed Caco-2 and SW48 cells were determined and compared to vector control groups after 48 h incubation with RGS. The data are presented as the mean ± standard error of the mean for 3 different experiments performed in triplicate. **P < 0.01; n.s., not significant.

To evaluate the effect of RGS on CRC cell viability, 7 CRC cell lines (SW48, Caco-2, DLD1, HCT116, LOVO, SW620, and SW480) with a different *RAS* mutational status were treated with RGS at concentrations ranging from 0 to 1,000 nM or 0 to 20 μM for 96 h or 48 h, respectively. As shown in **Figure 1A and 1B**, RGS exhibited a cytotoxic effect against all 7 CRC cell lines, but the sensitivity to RGS varied greatly in different cell lines. Compared to the other 5 *KRAS*-mutant CRC cell lines, SW48 and Caco-2 cells, which harbored the wild-type *RAS* genes, were relatively resistant to RGS, and even when treated with the maximum concentration of RGS (20 μM for 48 h and 1,000 nM for 96 h), the cell viability was greater than 50% (58.6% and 55.4% for 48 h, 65.7% and 70.6% for 96 h, respectively). We also used a clone formation assay to determine the inhibitory effect of RGS on cell proliferation, and found that after 2 weeks of incubation, there were more remaining clones of SW48 and Caco-2 than the other 5 *KRAS* mutant CRC cell lines (**Supplementary Figure S1A**). The differences in the remaining cells after 24 h of incubation of 1–5 μM RGS between SW48/Caco-2 and DLD1/HCT116 also showed relative resistances to RGS in the Caco-2 and SW48 cells (**Supplementary Figure S1B**).

Subcutaneous xenograft tumor models were used to confirm the differential activity of RGS in SW48 (*RAS* wild-type) and DLD1 (*KRAS G13D* mutation) *in vivo*, and tumor-bearing nude mice were randomly assigned to receive PBS or RGS. RGS treatment had little effect on body weight in both SW48 and DLD1 xenograft mice (**Supplementary Figure S1C**). Significant inhibition of tumor growth was observed in RGS-treated DLD1 xenograft mice (but not in the SW48 xenograft models), when compared to the PBS-treated group (**Figure 1C**). At the end of the experiment, the average tumor weight was significantly greater in the DLD1 PBS-treated group than in the RGS-treated mice (*P* < 0.01); in the SW48 xenograft models, there was no significant difference in tumor weight between the PBS-treated and RGS-treated mice (**Figure 1D**). Together, these *in vivo* and *in vitro* experiments suggested that the anti-tumor effect of RGS was probably dependent on the presence of KRAS mutations.

To determine the role of *KRAS* mutations in the sensitivity to RGS, Caco-2 and SW48 cell lines stably expressing His-tagged mutated KRAS (G12D, G12V, and G13D) were established, in which the RAS downstream signaling molecules were activated (**Supplementary Figure S1F**). Compared to the vector control cell lines, transduction of the mutant *KRAS* gene into Caco-2 and SW48 cells increased their sensitivities to RGS (**Figure 1E and 1F**).

Previously, Reddy et al.^8^ reported that RGS induced cell death in cancer cells, but with minimal cytotoxicity in normal cells. In our study, we incubated immortalized colon epithelial cells (CCD841CoN) and colon fibroblasts (CCD18Co) with high concentrations of RGS (200 nM and 1,000 nM) for 96 h, similar to the previous treatment of the resistant CRC cell lines (SW48 and Caco-2). The results showed that cell viabilities of CCD841CoN and CCD18Co were 79.3% and 94.0% at 200 nM, and 74.3% and 88.7% at 1,000 nM, respectively (**Supplementary Figure S1G**).

Taken together, RGS decreased CRC cell viability in a dose-dependent manner, while having less effect on immortalized colon epithelial cells/fibroblasts *in vitro*. Moreover, we confirmed that *KRAS*-mutant CRC cell lines were relatively more sensitive to RGS treatment.

### RGS induced mitochondria-related apoptosis and mitotic arrest in KRAS-mutant DLD1 and HCT116 cells

**Figure 2:**
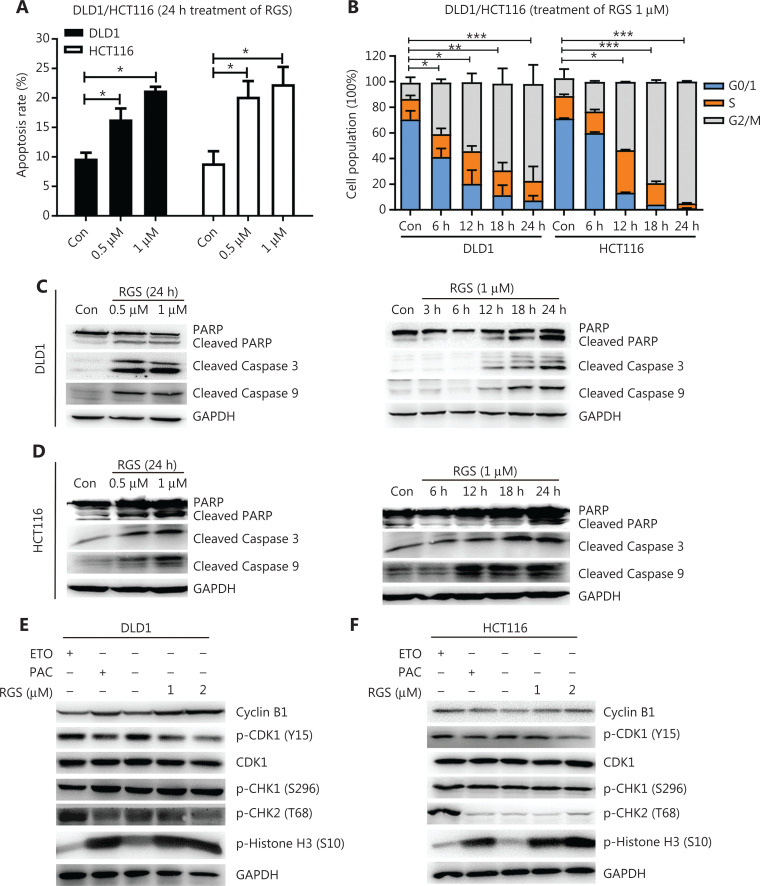
Rigosertib (RGS) induced apoptosis and mitotic arrest in *KRAS*-mutant DLD1 and HCT116 cells. DLD1 and HCT116 cells were treated with or without different concentrations of RGS for 24 h, and apoptosis was determined by flow cytometry after annexin V and propidium iodide staining (A). DLD1 and HCT116 cells treated at various times with RGS were examined for cell-cycle arrest by flow cytometry (B). DLD1 and HCT116 cells were treated with 0.5/1 μM RGS for 24 h or with 1 μM for various times prior to lysis. Apoptosis-related protein markers were examined as indicated (C, D). DLD1 and HCT116 cells were incubated with RGS, etoposide, and paclitaxel for 12 h, and cell cycle progression-associated markers were analyzed by Western blot (E, F). Error bars represent the mean ± standard error of the mean obtained from 3 independent experiments. *P < 0.05; **P < 0.01; ***P < 0.001; Con, control; PARP, poly (ADP-ribose) polymerase; GAPDH, glyceraldehyde 3-phosphate dehydrogenase.

We then analyzed the effects of RGS in modulating apoptosis in *KRAS*-mutant DLD1 and HCT116 CRC cells. Annexin-V/propidium iodide staining and flow cytometry were conducted to determine apoptosis. **Figure 2A and Supplementary Figure S2B** show that incubation with RGS for 24 h significantly induced apoptosis in both DLD1 and HCT116 cells. Moreover, apoptosis-related proteins, such as cleaved caspase-3, cleaved caspase-9, and cleaved poly (ADP-ribose) polymerase (PARP), increased in a time- and dose-dependent manner after RGS treatment (**Figure 2C and 2D**). Furthermore, the nuclei of RGS-treated cells showed “apoptosis-like” condensed chromatin, with a brighter appearance with shrunken and fragmented nuclei, when compared to the untreated cells (**Supplementary Figure S2A**). Because caspase-9 has been reported to be the central enzyme controlling mitochondrial related apoptosis^20^, its activation in RGS-treated cells indicated that RGS might modulate mitochondrial apoptosis. We therefore separated cytoplasmic and mitochondrial fractions of RGS-treated DLD1 and HCT116 cells (**Supplementary Figure S2C**), and found that the proapoptotic protein, Bcl-2-associated X protein (Bax), was significantly downregulated in the cytoplasm after 24 h of exposure to RGS, and cytochrome *c* (Cyt c) was significantly released from the mitochondria to the cytoplasm.

For cell cycle synchronization, DLD1 and HCT116 cells were incubated in a serum-free medium for 48 h, and then the 2 cell lines were treated with RGS for 0–24 h. The effect of RGS on cell cycle progression was assessed by flow cytometry (**Figure 2B**). The results showed a time-dependent G2/M cell cycle block (enhanced numbers of cells with 4N DNA content) in DLD1 and HCT116 cells. Next, we checked the morphological changes of RGS-treated DLD1 and HCT116 cells using May-Grünwald-Giemsa staining (**Supplementary Figure S2D**), and calculated the percentage of mitotic cells (mitotic index, **Supplementary Figure S2E**)^21^. The mitotic indices of DLD1 and HCT116 cells after RGS treatment were increased, when compared to the controls. To confirm the mitotic arrest activity of RGS, we tested protein markers, such as cyclin B1, CDK1, p-CDK1 (Y15), and p-Histone H3 (S10), related to G2/M arrest in RGS-treated DLD1 and HCT116 cells using Western blot. Etoposide and paclitaxel were used as positive controls for G2 and M phase arrests, respectively. As a classic inhibitor of topoisomerase II, etoposide induced DNA damage with elevation of p-CHK2 (T68) in DLD1 and HCT116 cells. Moreover, RGS and paclitaxel treatment activated the CDK1/cyclin B1 complex by inhibiting p-CDK1 (Y15) with elevated levels of cyclin B1. Combined with the increased phosphorylation of histone H3 at Ser10, a distinct biomarker of mitosis, we concluded that RGS induced mitotic arrest, not G2 arrest in DLD1 and HCT116 cells (**Figure 2E and 2F**).

### RGS disrupted EGF-induced RAS/MEK/ERK signaling independent of cellular ROS generation

**Figure 3:**
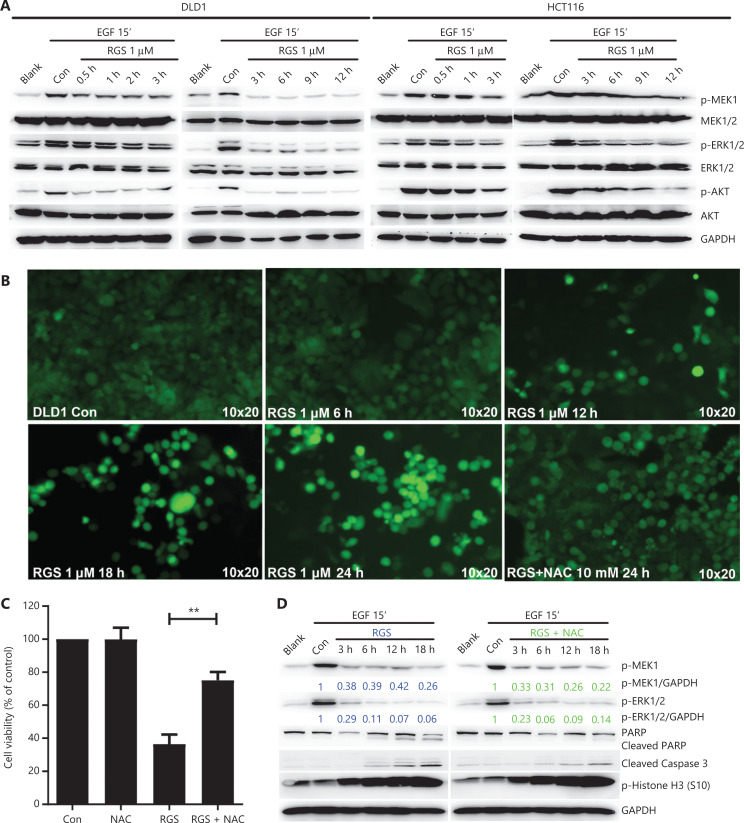
Rigosertib (RGS) interfered with the epidermal growth factor (EGF) receptor-induced RAS signaling pathway, and inhibition of RAS/MEK/ERK signaling was independent of cellular oxidative stress. DLD1 and HCT116 cells were serum starved overnight and treated as indicated with dimethyl sulfoxide or 1 μM RGS prior to stimulation with EGF for 15 min then lysed. Lysates were examined for MEK, pMEK, ERK, pERK, AKT, and pAKT levels and glyceraldehyde 3-phosphate dehydrogenase (loading control) using Western blot (A). DLD1 cells treated at various times with 1 μM RGS, with or without pretreatment with 10 mM of the antioxidant, N-acetylcysteine (NAC), were stained with DCFH-DA (a cellular reactive oxygen species probe) and examined using a fluorescence microscope at 10 × 20 times magnification (B). The cell viability of DLD1 cells after 18 h treatment with dimethyl sulfoxide, N-acetylcysteine (NAC), RGS, or NAC + RGS was determined (C). DLD1 cells were treated as indicated with RGS or NAC + RGS prior to stimulation with EGF, and cell lysates were examined for activated pMEK and pERK levels, apoptosis-related marker levels (PARP and cleaved caspase 3), and the mitotic arrest marker level (p-Histone H3) (D). Error bars represent the mean ± standard error of the mean obtained from 3 independent experiments. **P < 0.01.

In consideration of the critical role of RGS in regulation of RAS/MEK/ERK and PI3K/AKT signaling in *KRAS*-mutant cancer cells as reported by Athuluri-Divakar et al.^11^ and Ritt et al.^12^, we evaluated the levels of activated pMEK, pERK, and pAKT in DLD1 and HCT116 cells after 0.5–12 h of RGS incubation (**Figure 3A**). Similar to the results of Amodio et al.^22^, EGF treatment rapidly activated the ERK cascade and PI3K/AKT signaling in these 2 *KRAS*-mutant CRC cell lines, as evidenced by high levels of activated pMEK, pERK, and pAKT. Additionally, we found that EGF-induced RAS/MEK/ERK and PI3K/AKT signaling were rapidly and sustainably disrupted after 0.5–12 h of RGS treatment, with only low levels of pMEK, pERK, and pAKT observed.

To determine whether RGS-induced RAS/MEK/ERK inhibition was an oxidative stress dependent process as reported by Ritt et al.^12^, we first evaluated the generation and localization of ROS by 2′–7′ dichlorofluorescin diacetate (DCFH-DA) staining after RGS treatment. Similar to the results of Chapman et al.^23^, whereby RGS induced mitochondrial depolarization and accumulation of ROS, which in turn activated the oxidative stress-dependent apoptosis pathway, we found that the ROS levels were significantly elevated after 12–24 h of treatment of RGS in DLD1 cells, yet when cells were co-treated with RGS and the antioxidant N-acetylcysteine (NAC), ROS accumulation was reduced to near background levels (**Figure 3B**). Moreover, DCFH-DA (the specific sensor for cellular ROS generation) and Mito-Tracker co-staining indicated that mitochondria were the major source of ROS production in DLD1 cells following exposure to RGS (**Supplementary Figure S3A**). Pretreatment with the ROS scavenger, NAC, restored DLD1 cell viability and reduced apoptosis-related protein levels (cleaved PARP and cleaved caspase-3) after RGS treatment, but the RGS-induced biomarker of mitotic arrest (phosphorylation of histone H3 at Ser10) was largely unaffected (**Figure 3C and 3D**). Combined with the results shown in **Figure 2**, these findings suggested that mitotic arrest caused by RGS may be the initiating stress that promoted mitochondrial ROS production and then mediated ROS-dependent apoptosis. However, in our experiments, pretreatment with NAC did not impair the effect of RGS in inhibiting RAS downstream activation of MEK and ERK (**Figure 3D, Supplementary Figure S3B**).

In summary, RGS disrupted EGF-induced RAS/MEK/ERK and PI3K/AKT signaling and induced oxidative stress-dependent apoptosis in *KRAS*-mutant CRC cells. However, inhibition of the RAS/MEK/ERK pathway was independent of cellular ROS generation.

### RGS inhibited RAS-mediated-signaling and suppressed tumor growth in KRAS-mutant colorectal cancer patient-derived xenograft models

**Figure 4:**
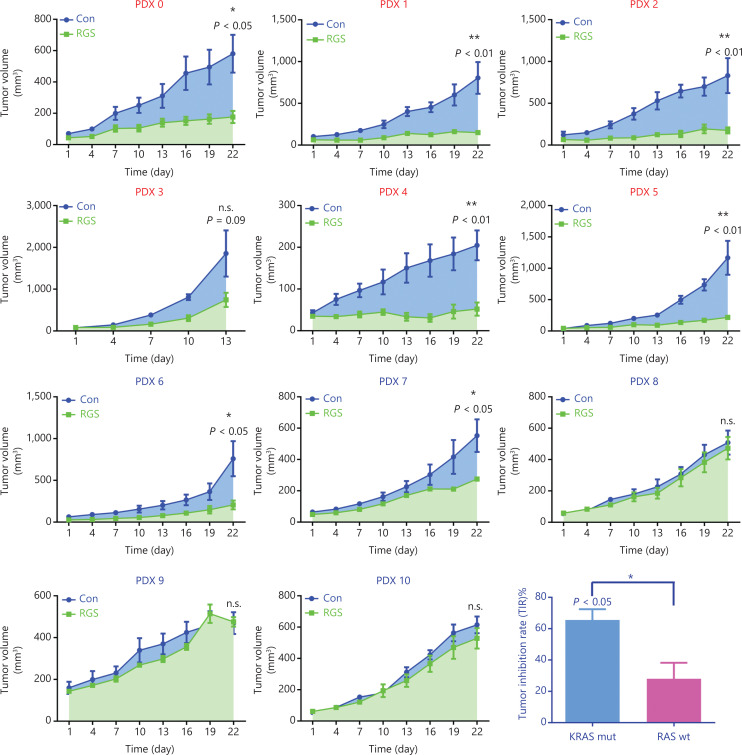
Rigosertib (RGS) suppressed tumor growth in *KRAS*-mutant colorectal cancer patient-derived xenograft models more efficiently than in *RAS* wild-type (wt) patient-derived xenograft (PDX) models. A stable PDX bank was established from 11 patients with *KRAS*-mut (PDX 0–5) or *RAS* wt (PDX 6–10) CRC. After the subcutaneous tumors reached 50–100 mm^3^, the animals were randomly assigned to receive phosphate-buffered saline or RGS by intraperitoneal injection (*N* = 5 per group) for 3 weeks. The tumor size and mice weight were measured every 3 days. At termination, isolated tumors were weighed, and the anti-tumor activity was determined by the tumor inhibition rate (TIR). TIR = (1 - W_T_/W_C_) × 100%; W_T_ = tumor weight of the RGS-treated group, W_C_ = tumor weight of the PBS-treated group). Tumor volumes are expressed as the mean ± standard error of the mean. *P < 0.05; **P < 0.01; n.s., not significant.

Patient-derived xenograft (PDX) models were used to investigate the anti-tumor effects of RGS in *RAS* wild-type and *KRAS*-mutant CRC patients. Eleven patients (6 with *KRAS*-mutant and 5 with *RAS* wild-type CRC) with tumor specimens were enrolled in this study. The baseline information including tumor sites, TNM stage, pathological differentiation, and *RAS* mutation status are shown in **Supplementary Table S1**. After the PDX models were established, animals were randomly assigned to be injected by PBS or RGS. According to the tumor growth curves shown in **Figure 4**, RGS showed a substantial anti-tumor effect in 63.6% (7/11) of PDX models, with the tumor inhibition rate (TIR) ranging from 4.11% to 83.2% (**Supplementary Table S1**). In the *KRAS*-mutant subgroup, 83.3% (5/6) of PDX models were sensitive to RGS treatment, but the response to RGS was only 40% (2/5) in the *RAS* wild-type subgroup (**Figure 4**). In addition, the TIR was significantly higher in the *KRAS*-mutant PDX subgroup (41.2%–83.2%) than in the *RAS* wild-type group (4.11%–64.4%) (*P* < 0.05). The detailed tumor growth curves, mice weight change curves, and tumor weights at the end of the experiments are shown in **Supplementary Figures S4 and S5**. In all 11 PDX models, there was no evidence of drug toxicity, as determined by a change in the body weights of mice (**Supplementary Figures S4 and S5**, column 2).

Collectively, as a novel antineoplastic drug, the anti-tumor effect of RGS in CRC was verified in our experiments. More importantly, RGS was more potent and efficacious in *KRAS*-mutant CRC than in *RAS* wild-type CRC.

**Figure 5:**
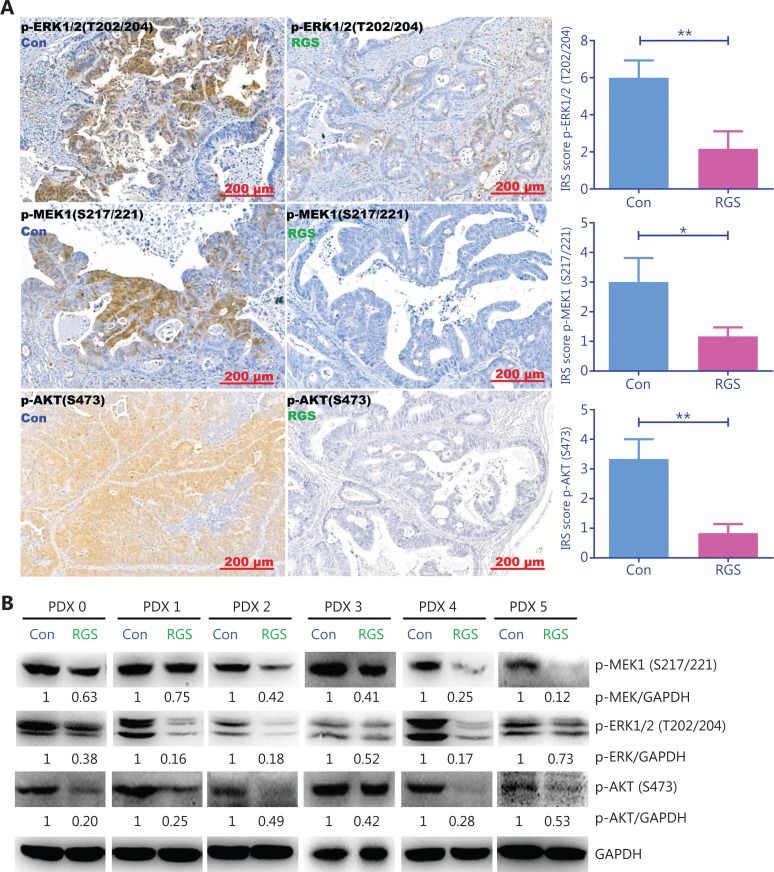
Rigosertib (RGS) inhibited RAS-mediated-signaling in *KRAS*-mutant colorectal cancer patient-derived xenograft tumors. After intraperitoneal RGS treatment, phosphorylation levels of ERK, MEK, and AKT in 6 subcutaneous tumors of *KRAS*-mutant CRC patient-derived xenograft (PDX) models were evaluated by immunohistochemical staining, and the immunoreactive scores of phosphorylated protein staining were calculated (A). The data are presented as the mean ± standard error of the mean for 3 different experiments performed in triplicate. *P < 0.05; **P < 0.01. The levels of MEK, ERK, and AKT phosphorylation in *KRAS*-mutant PDX tumors were examined by Western blot, and levels of pMEK, pERK, and pAKT were quantitated by ImageJ software (B).

To confirm the effect of RGS on the suppression of RAS-mediated signaling, we examined the levels of MEK, ERK, and AKT phosphorylation in *KRAS*-mutant patient-derived xenograft tumors. As expected, there was robust phosphorylation of MEK, ERK, and AKT in the control *KRAS*-mutant tumors, but the phosphorylation of these proteins was markedly inhibited in RGS-treated tumors (**Figure 5A and 5B**), indicating that RGS treatment reduced the level of RAS-mediated signaling.

### RGS was efficacious in patient-derived xenograft models derived from a KRAS-mutant colorectal cancer patient who was resistant to modified FOLFOX6 + bevacizumab and FOLFIRI + bevacizumab

**Figure 6:**
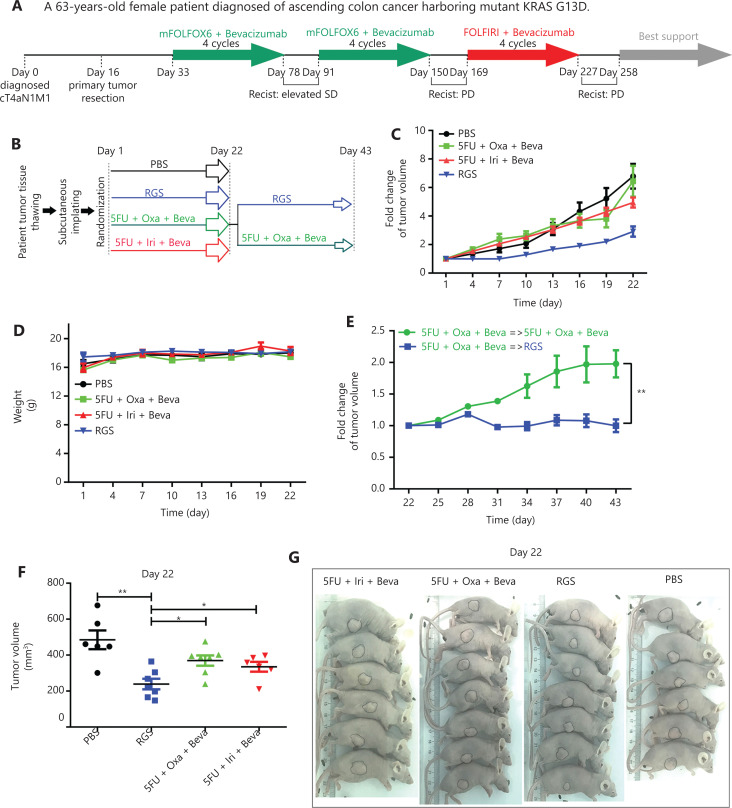
Rigosertib (RGS) was effective in patient-derived xenograft (PDX) models from a *KRAS*-mutant colorectal cancer patient who was resistant to modified FOLFOX6 (mFOLFOX6) + bevacizumab and FOLFIRI + bevacizumab treatment. A brief medical history of the *KRAS*-mutant chemotherapy-resistant colorectal cancer patient was provided. On day 16, fresh surgical specimens were obtained and implanted subcutaneously in nude mice. Once the subcutaneous tumors grew to 500 mm^3^, the tumors were harvested and cryopreserved in a refrigerator (P0) (A). When this patient was enrolled retrospectively in this study, the frozen human tumor tissue was revived and replanted to establish PDX models (P1). The detailed experimental schedule involving grouping and treatment duration are shown in B. Tumor growth curves from day 1 to day 22, following phosphate-buffered saline, RGS, fluoropyrimidine (5-FU) + oxaliplatin + bevacizumab, and 5-FU + irinotecan + bevacizumab treatment (C). No significant difference in mice weight among these 4 groups was found (D). Tumor growth curves from day 22 to day 43 of 2 subgroups of 5-FU + oxaliplatin + bevacizumab mice (a group that received RGS and a group that received the originally prescribed drugs) are shown (E). Tumor volumes were calculated on day 22 in these 4 groups (F). Representative images of gross morphology when the mice were anesthetized on day 22 (G). Tumor volumes are expressed as a mean ± standard error of the mean. *P < 0.05; **P < 0.01.

To compare the anti-tumor effect of RGS and fluoropyrimidine (5-FU)/irinotecan/oxaliplatin-based standard chemotherapy in advanced CRC, PDX models derived from a *KRAS*-mutant patient who was primarily resistant to standard therapy were used in our study. A 63-year-old female patient was diagnosed as having a *KRAS*-mutant ascending colon cancer with distant metastasis (cT4aN1M1). After primary tumor resection on day 16, she received 8 cycles of modified FOLFOX6 + bevacizumab and 4 cycles of FOLFIRI + bevacizumab systemic treatment, and the tumor burden showed continuous progression after these 2 standard strategies according to the Response Evaluation Criteria in Solid Tumors criteria (**Figure 6A**). After the patient was retrospectively enrolled and the corresponding PDX models were established, nude mice were divided into 4 groups that received PBS, RGS, 5-FU + oxaliplatin + bevacizumab, or 5-FU + irinotecan + bevacizumab for 3 weeks. On day 22, the 5FU + oxaliplatin + bevacizumab group was further divided into 2 subgroups; 1 group received RGS injection until day 43, while the other group continued to receive the originally prescribed drugs. The detailed experimental schedule is shown in **Figure 6B**.

According to the tumor growth curves shown in **Figure 6C**, RGS significantly inhibited tumor growth compared to PBS treatment; moreover, tumor growth was slower in the RGS-treated group than in the 5-FU + oxaliplatin + bevacizumab and 5-FU + irinotecan + bevacizumab groups. Tumor volumes calculated on day 22 also showed that the tumor volume in RGS-treated mice was smaller than that in the other 3 groups (**Figure 6F**). Additionally, there was no significant difference in mice weight during treatment (**Figure 6D**). As shown in **Figure 6E**, compared to the originally prescribed drugs, RGS treatment instead of 5-FU + oxaliplatin + bevacizumab on day 22 suppressed the resistant tumor growth.

In summary, in this *KRAS*-mutant and continuously progressive CRC case, RGS was more efficacious than the combination of fluoropyrimidine/irinotecan/oxaliplatin and bevacizumab.

## Discussion

In this study, we found that RGS inhibited cell viability in CRC cell lines, and that this anti-tumor effect in *KRAS*-mutant CRC cells was significantly stronger than that in *RAS* wild-type cells. This differential therapeutic effect in *KRAS*-mutant and *RAS* wild-type CRC was also confirmed using our PDX models. We then found that RGS induced mitotic arrest and oxidative stress-dependent apoptosis in a time-dependent and dose-dependent manner in *KRAS*-mutant CRC cells. Moreover, RGS treatment disrupted RAS/MEK/ERK and PI3K/AKT signaling in *KRAS*-mutant CRC cell lines and *KRAS*-mutant PDX tumor specimens. Finally, in PDX models derived from a *KRAS*-mutant, chemotherapy-resistant CRC case, RGS decreased subcutaneous tumor growth more effectively than the combination of 5-FU, irinotecan/oxaliplatin, and bevacizumab treatment, which is the first-line clinical treatment for *KRAS*-mutant mCRC patients. In brief, our research confirmed that RGS exhibited a dramatic and selective anti-tumor effect in *KRAS*-mutant colorectal cancer when compared with *RAS* wild-type CRC, and that this anti-tumor effect was associated with RGS-induced inhibition of RAS signaling.

*KRAS* is one of the most frequently mutated oncogenes in CRC, and mutant KRAS oncoprotein can activate downstream RAF/MEK/ERK and PI3K/AKT signals, which are responsible for cancer cell proliferation, survival, and evasion of apoptosis. Because of the lack of druggable targets on the surface of RAS, the development of compounds that directly target mutant KRAS has been largely unsuccessful^24^. Recently, this obstacle was partly overcome by the development of covalent KRAS G12C-specific inhibitors, such as AMG510 and MRTX849^25,26^, but this therapy targeting the KRAS G12C mutation could not benefit all patients. Yang et al.^27^ reported that the *KRAS G12C* gene mutations only accounted for 2.7%–5.6% of total *KRAS*-mutant CRC patients. Because of the inefficiency of targeting single RAS downstream effectors (such as MEK and PI3K)^28,29^, other investigators have attempted to use combinational blockage of MEK and PI3K to block the RAS signaling pathway. They found that blockage of MEK and PI3K suppressed tumor growth in *KRAS*-driven lung cancer mouse models^30^. However, the striking toxicity of this combinational therapy limited its clinical applicability^31^. In our study, we showed that RGS, a reported small molecular RAS signaling disruptor, had a selective anti-tumor effect in *KRAS*-mutant CRC. Unlike AMG510 and MRTX849, which target the specific *KRAS G12C* mutation, the anti-tumor effect of RGS existed in CRC harboring multiple types of *KRAS* mutations. In *KRAS* mutant CRC PDX models responding to RGS treatment, the *KRAS* mutation types included *KRAS* *G13D*, *G12A*, *G12V*, and *G12S*. Along with its surprising curative effect, RGS showed minimal cytotoxicity in immortalized colon epithelial cells and fibroblasts, and there was no evidence of drug toxicity as determined by changes in mice body weight in our *in vivo* experiments. Considering the dramatic therapeutic effect and minimal drug toxicity, targeting RAS signaling by RGS may be a promising clinical treatment for *KRAS*-mutant CRC.

RGS has been studied for more than 10 years. Initially, RGS was thought to be an inhibitor of PLK1 kinase, which induced mitotic arrest characterized by spindle abnormalities^32^, but Steegmaier et al.^33^ showed that RGS did not directly inhibit PLK1 activity. Other mechanisms suggested that RGS disrupted RAS/MEK/ERK and PI3K/AKT signaling, but there was a distinct difference in these hypotheses. Athuluri-Divakar et al.^11^ suggested that RGS blocked RAS/effector interactions and directly inhibited RAS/MEK/ERK and PI3K/AKT signaling. When compared to rapid inhibition of PI3K/AKT (2 h), Ritt et al.^12^ reported that RGS inhibited the RAS/MEK/ERK pathway only at longer time points (18 h). Based on this unexpected phenomenon, the authors investigated the effect of RGS on regulating the RAS/MEK/ERK pathway and found that RGS could suppress RAS/MEK/ERK signaling indirectly by the oxidative stress-dependent phospho-inhibition circuit. In the present study, we determined whether RGS inhibited the RAS downstream signaling pathway. Consistent with the results of Ritt et al.^12^, we found that RGS quickly decreased the p-AKT levels in *KRAS*-mutant CRC cells after 3 h of incubation, which was consistent with the results reported by Prasad et al.^34^ and Chapman et al.^23^ for hematological malignant tumors. However, in contrast to the findings of Ritt et al.^12^, we also found that RGS rapidly downregulated p-MEK and p-ERK levels activated by EGF stimulation after 3 h of incubation.

Ritt et al.^12^ evaluated p-MEK and p-ERK levels in HeLa cells incubated in full culture medium containing serum; however, in our study, the *KRAS*-mutant CRC cells underwent additional serum starvation overnight prior to RGS treatment and EGF stimulation. Jiang et al.^35^ suggested that extracellular signal deprivation by serum starvation significantly decreased background ERK/MAPK activation. It is likely that because of the elimination of baseline ERK/MAPK activation noise, these CRC cells were more sensitive to subsequent EGF stimulation, and the ERK/MAPK signaling variation could have been detected with more sensitivity in our studies.

In our research and studies reported by Liu et al.^36^ and Ritt et al.^12^, long-term RGS treatment (12–24 h) induced cellular oxidative stress; however, we found that the initial inhibition of MEK/ERK signaling (1–3 h) usually occurred ahead of ROS generation, and pretreatment with a ROS scavenger could not restore the decrease in p-MEK and p-ERK levels. Together, combined with decreased levels of MEK, ERK, and AKT phosphorylation in RGS treated *KRAS*-mutant PDX tumors, our study confirmed that RGS was an efficient RAS signaling disruptor; but its inhibition of RAS/MEK/ERK pathway was probably independent of cellular ROS generation. Perhaps a stress-induced checkpoint to block RAS/MEK/ERK signaling does exist, as reported by Ritt et al.^12^, but that might be a secondary effect of long-term incubation after multiple RGS-induced and devastating cellular processes were activated. After 18 h of incubation, RGS-incubated cancer cells did not maintain normal cell morphology, and produced vast amounts of cell fragments because of RGS-activated apoptosis (data not shown).

In the present study, we focused on the translational potential of RGS in *KRAS*-mutant colorectal cancer treatment; the selective anti-tumor effect of RGS in *KRAS* mutant CRC was comprehensively studied, for the first time, in models ranging from cancer cell lines to patient-derived xenograft models. Additionally, while the direct target of RGS remains controversial, our research verified that RGS significantly disrupted activated RAS signaling in both CRC cell lines and patient’s tumor tissues, and that this provided a theoretical basis for the potential clinical application of RGS to treat *KRAS*-mutant CRC. However, some issues remain to be solved. First, as a multiple kinase inhibitor, the reported mechanism of RGS included PLK1 inhibition, PI3K/AKT inhibition, blocking RAS/MEK/ERK signaling, and even destabilizing microtubules^37^. In our study, we confirmed that RGS inhibited RAS signaling, but we did not investigate PLK1 kinase activity or microtubule dynamics in RGS-treated CRC samples; thus, our results could not eliminate the contribution of other signaling pathways. Second, the *KRAS* mutation profiles in CRC cell lines and PDX tumors in our studies did not include the *KRAS G12C* mutation, which was targeted by KRAS G12C inhibitors (AMG510 and MRTX849); therefore, we could not compare the curable effect of RGS and these 2 promising compounds.

## Conclusions

Taken together, our findings showed that RGS, a small molecular RAS signaling disruptor, had a selective anti-tumor effect and no obvious toxicity in *KRAS* mutant CRC. Further studies are therefore warranted for clinical evaluation of the use of RGS in the treatment of CRC.
