# Supplementary material for: Efficacy of rigosertib, a small molecular RAS signaling disrupter for the treatment of KRAS-mutant colorectal cancer
Source: Cancer Biol Med. 2021 Aug 4;19(2):213–28. doi: 10.20892/j.issn.2095-3941.2020.0532 (PMC8832955; doi:10.20892/j.issn.2095-3941.2020.0532)
Supplement: Supplementary file 1 [file cbm-19-213-s001.pdf]

Supplementary materials

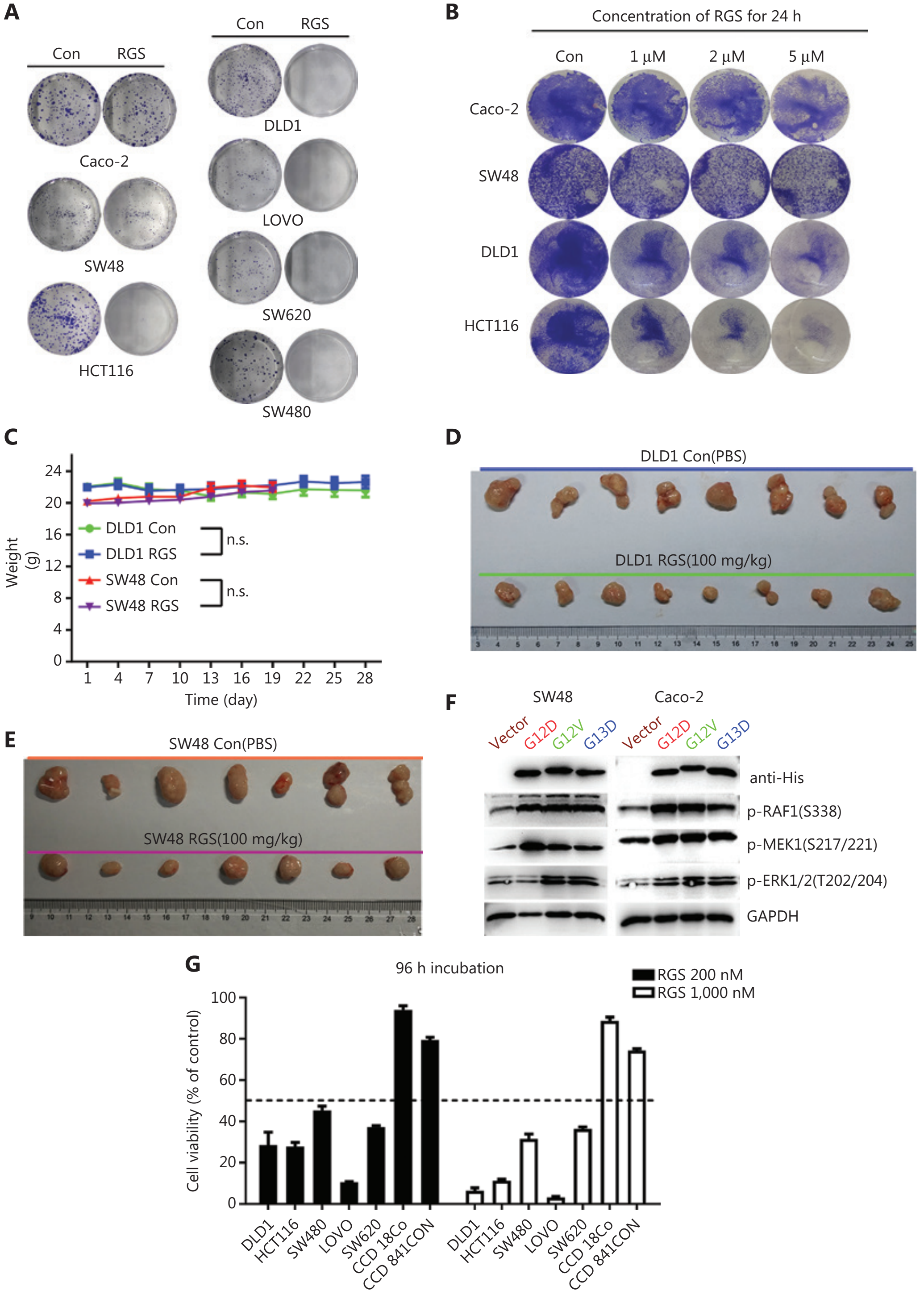

Figure S1 Related to **Figure 1**. Evaluation of the anti-cancer effect of rigosertib (RGS) in colorectal cancer (CRC) cell lines and immortalized colon epithelial cells/fibroblasts. Image of the clone formation assay with 2 *RAS* wild-type CRC cells (Caco-2 and SW48) and 5 *KRAS*-mutant CRC cells (DLD1, HCT116, SW480, SW620, and LOVO) after 2 weeks incubation with or without 50 nM RGS (A). Caco-2/SW48 and DLD1/HCT116 were treated with 1–5 μM RGS for 24 h, and the remaining cells were stained with Crystal Violet (B). In the DLD1 and SW48 xenograft models, RGS administration showed no significant effect on mice body weight in both DLD1 and SW48 xenograft mice (C). Representative images of

gross morphology at termination of the DLD1 and SW48 xenograft experiments (D, E). Phosphorylation levels of the downstream effectors of KRAS (p-RAF, p-MEK, and p-ERK) were evaluated by Western blot in His-tagged *KRAS G12D*-, *G12V*-, *G13D*-expressed Caco-2, and SW48 cells (F). Cell viability of DLD1, SW480, SW620, HCT116, LOVO, CCD841CoN, and CCD18Co cell lines after 96 h treatment with 200 nM and 1,000 nM RGS (G). Error bars represent the mean ± standard error of the mean obtained from 3 independent experiments. n.s., not significant.

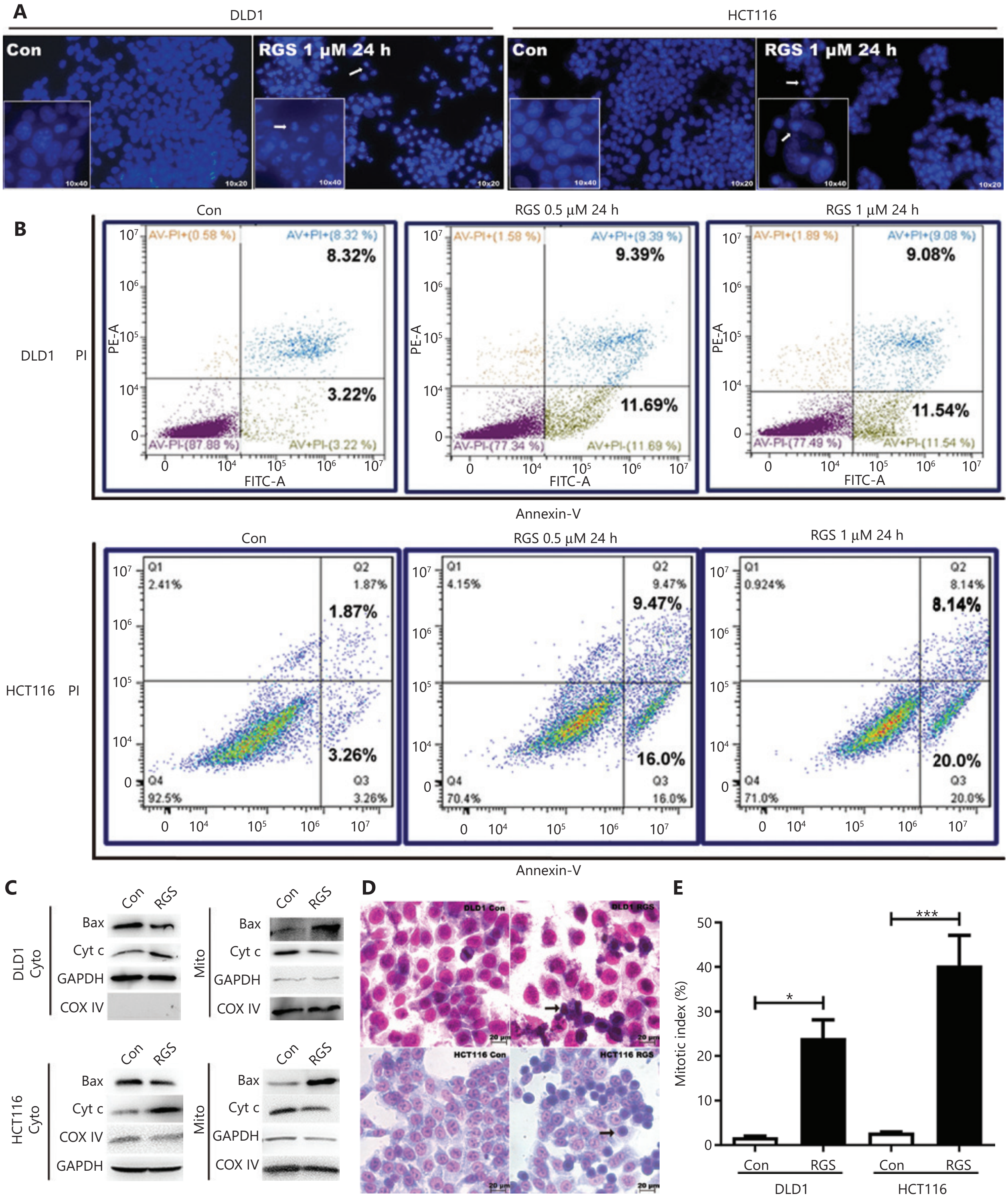

Figure S2 Rigosertib (RGS) induced mitochondria-related apoptosis and mitotic arrest in *KRAS*-mutant DLD1 and HCT116 cells. DLD1 and HCT116 cells were treated with 1 μM RGS for 24 h, and changes in nuclear morphology were detected after staining with 4′,6-diamidino-2-phenylindole and examining the cells by fluorescence microscopy at 10 × 20 and 10 × 40 times magnification. The shrunken and fragmented nuclei are indicated by white arrows (A). Representative images from flow cytometry of annexin V-propidium iodide-stained DLD1 and HCT116 cells after 24 h treatment with RGS (B). Levels of apoptosis-related proteins were determined by Western blot of cytoplasmic and mitochondrial fractions of DLD1 and HCT116 cells following a 24 h incubation with 1 μM RGS. Cytochrome c oxidase was the loading control for the mitochondrial fraction (C). Morphological changes in DLD1 and HCT116 cell lines following treatment with RGS. The cells were cultured with 1 μM RGS for 12 h, and the cytospin slides are indicated (May-Grünwald-Giemsa stain) (D). The percentages of mitotic cells (mitotic index) were calculated for RGS-treated and untreated DLD1 and HCT116 cells (DLD1 mitotic index change: 24.1 ± 4.06% *vs* 1.75 ± 0.24%, $P < 0.05$; HCT116 mitotic index change: 40.3 ± 9.58% *vs* 2.79 ± 0.25%, $P < 0.001$) (E). Error bars represent the mean ± standard error of the mean obtained from 3 independent experiments. *$P < 0.05$; ***$P < 0.001$; Con, control; GAPDH, glyceraldehyde 3-phosphate dehydrogenase; Bax, Bcl-2-associated X protein; Cyt c, cytochrome *c*.

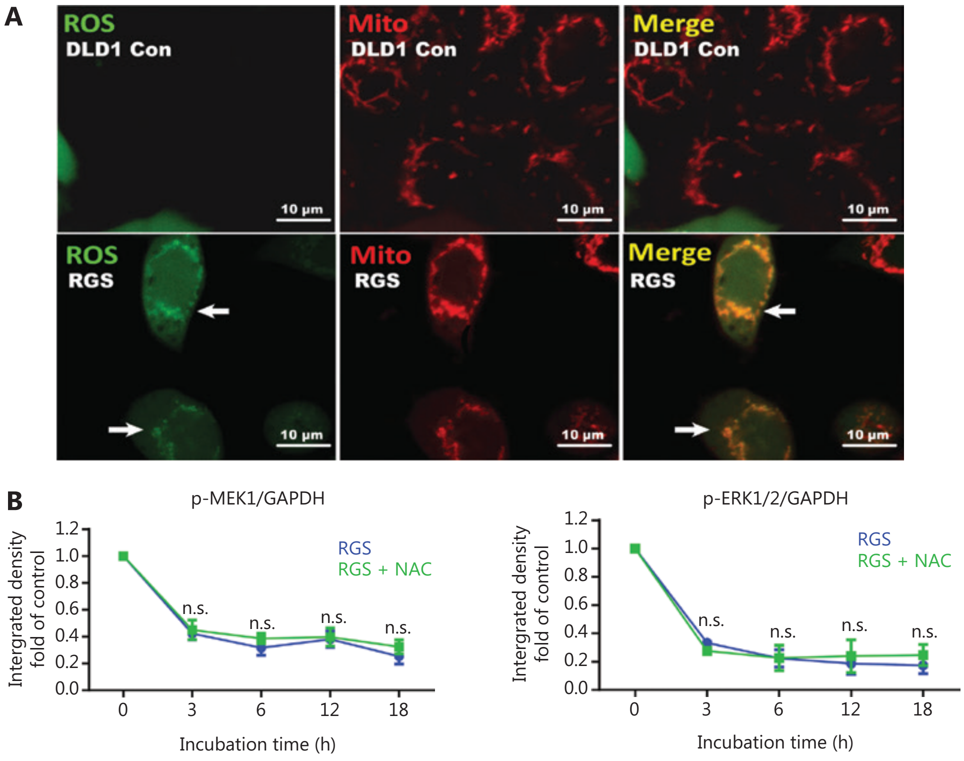

Figure S3 Related to Figure 3. Rigosertib (RGS) disrupted epidermal growth factor (EGF)-induced RAS/MEK/ERK signaling independent of mitochondrial reactive oxygen species (ROS) generation. DLD1 cells were treated with 1 μM RGS for 18 h, and then exposed to 10 μM 2′,7′ dichlorofluorescin diacetate (DCFH-DA) and 500 nM MitoTracker for 1 h. Localization of ROS was estimated by measuring DCFH-DA and MitoTracker fluorescence (A). DLD1 cells were treated as indicated with RGS or N-acetylcysteine + RGS prior to stimulation with epidermal growth factor, and the activated pMEK and pERK levels were quantified by ImageJ software. (B). Error bars represent the mean ± standard error of the mean obtained from 3 independent experiments. n.s., not significant.

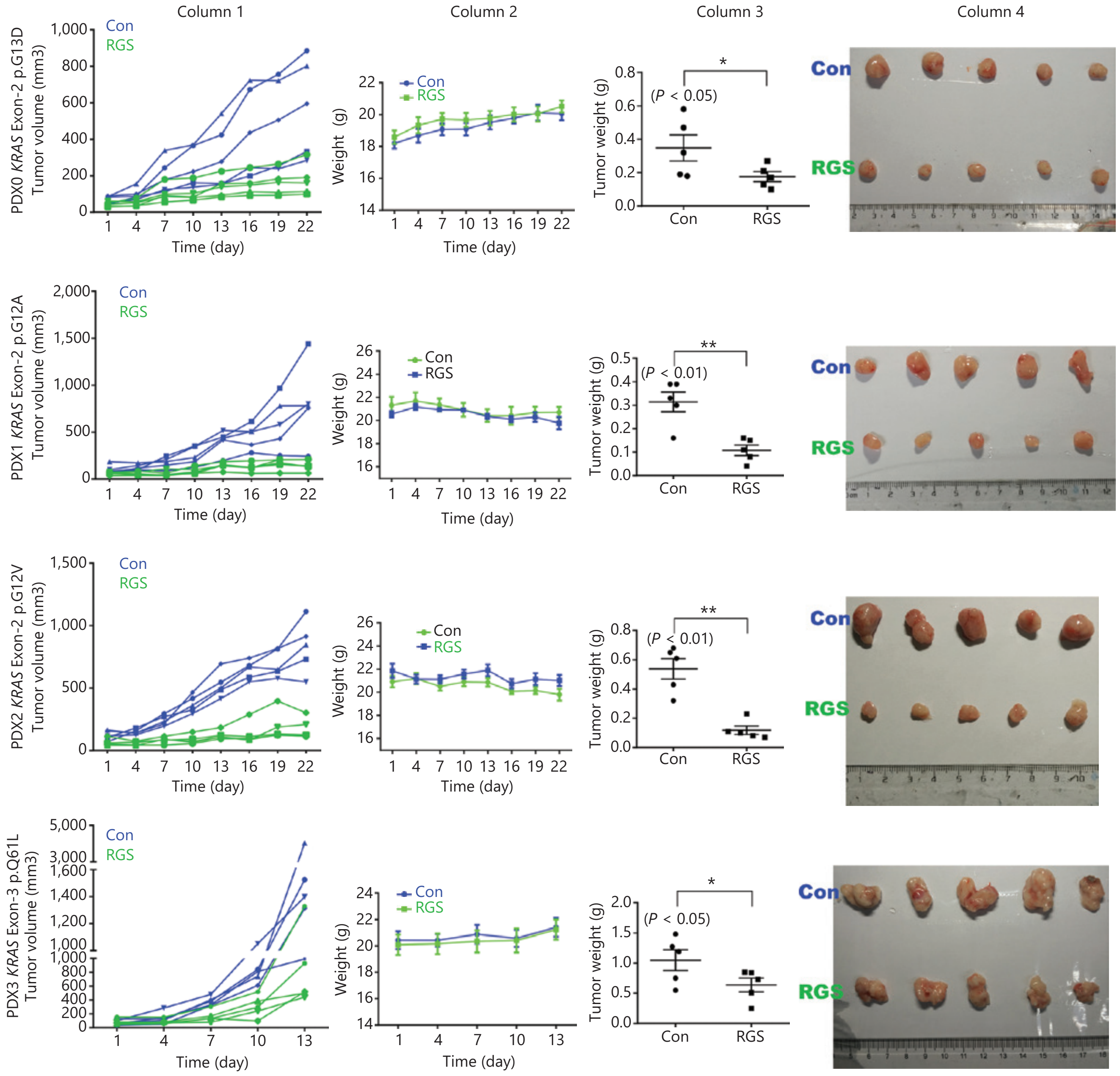

Figure S4 Continued

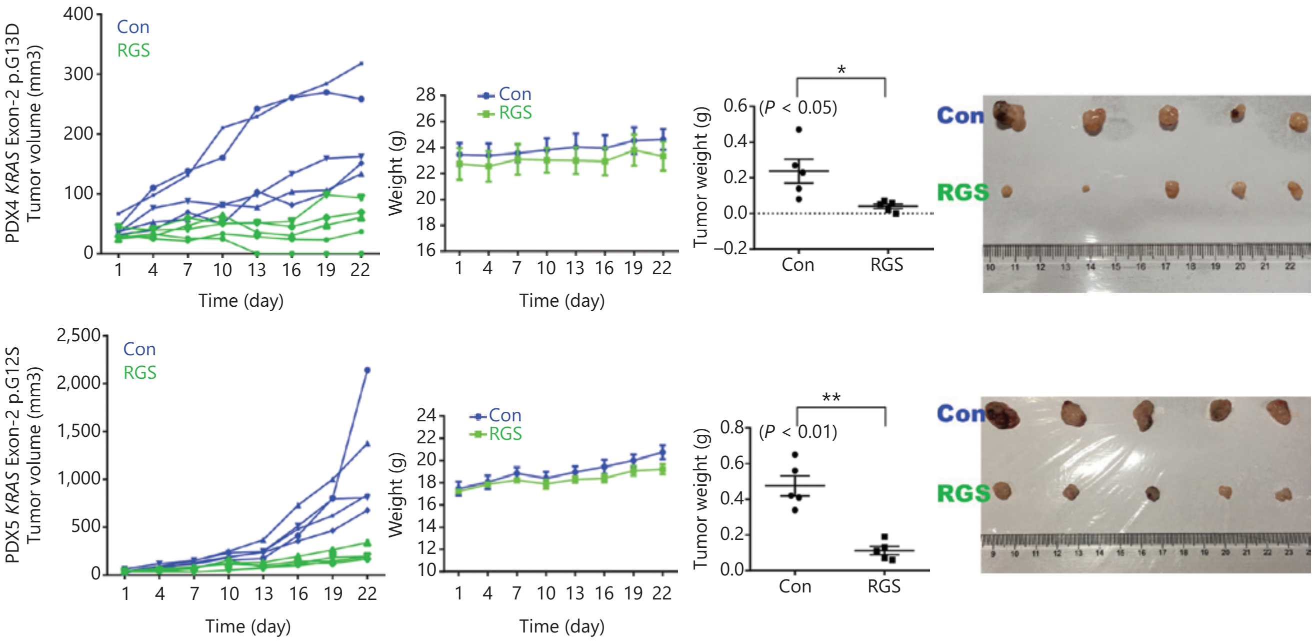

Figure S4 Detailed tumor growth curves, mice weight change curves, representative images of gross tumor morphology, and tumor weight information at termination in the *KRAS*-mutant patient-derived xenograft (PDX) models. Column 1. The detailed tumor growth curves of phosphate-buffered saline (PBS)-treated and RGS-treated groups in the *KRAS*-mutant PDX models (PDX0-PDX5). Column 2. There was no significant difference in mice weight between PBS-treated and RGS-treated animals in PDX0-PDX5. Column 3. The difference in tumor weight between PBS-treated and RGS-treated PDX models was calculated at the end of the experiments. Column 4. Representative images of gross tumor morphology. Error bars represent the mean ± standard error of the mean. *$P < 0.05$; **$P < 0.01$; Con, control.

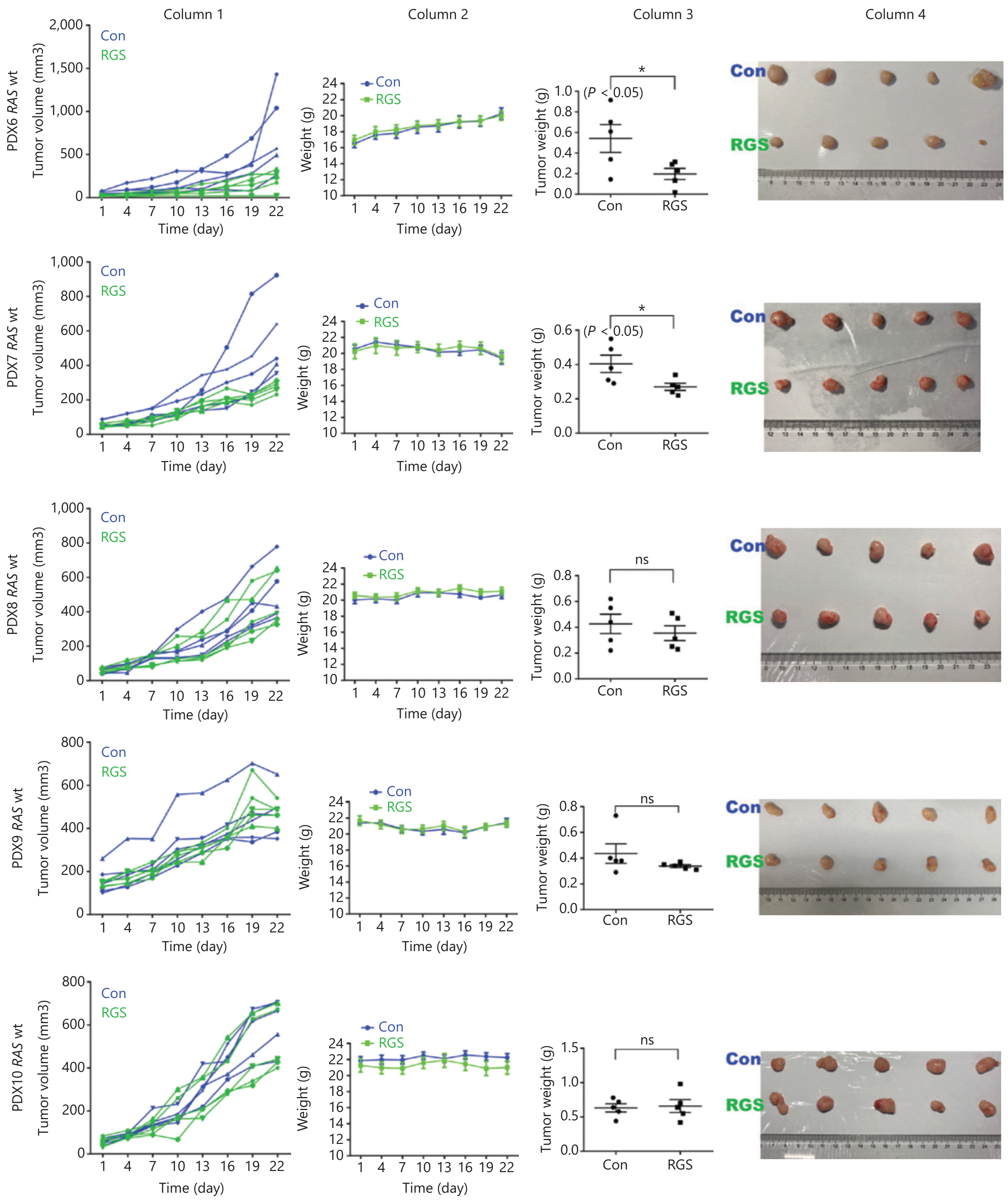

Figure S5 Detailed tumor growth curves, mice weight change curves, representative images of gross tumor morphology, and tumor weight information at termination in the *RAS* wild-type patient-derived xenograft (PDX) models. Column 1. The detailed tumor growth curves of phosphate-buffered saline (PBS)-treated and RGS-treated groups in the *RAS* wild-type PDX models (PDX6-PDX10). Column 2. There was no

significant difference in mice weight between PBS-treated and RGS-treated animals in PDX6-PDX10. Column 3. The difference in tumor weight between the PBS-treated and RGS-treated PDX models was calculated at the end of the experiments. Column 4. Representative images of gross tumor morphology. Error bars represent the mean ± standard error of the mean obtained. *P < 0.05; n.s., not significant.

Table S1 Patient characteristics, tumor pathological information, *RAS* status, and tumor inhibition rate (TIR) of patient-derived xenograft models

PDX	Age	Gender	Tumor site	Differentiation	TNM	*RAS* status	TIR (%)
**PDX0**	63	F	Right-side colon	Poorly	T3N2M1	*KRAS Exon-2 p.G13D*	50.5
**PDX1**	29	F	Left-side colon	Poorly	T4N2M0	*KRAS Exon-2 p.*G12A	64.0
**PDX2**	57	M	Rectum	Moderately	T4N2M0	*KRAS Exon-2 p.G12V*	78.1
**PDX3**	41	F	Rectum	Poorly	T4N1M0	*KRAS Exon-3 p.Q61L*	41.2
**PDX4**	63	M	Right-side colon	Moderately	T4N0M0	*KRAS Exon-2 p.G13D*	83.2
**PDX5**	60	M	Rectum	Well	T4N1M1	*KRAS Exon-2 p.G12S*	76.5
**PDX6**	64	M	Left-side colon	Moderately	T3N1M1	*Wild type*	64.4
**PDX7**	85	F	Right-side colon	Poorly	T4N2M0	*Wild type*	33.2
**PDX8**	56	M	Right-side colon	Moderately	T4N2M1	*Wild type*	17.0
**PDX9**	73	M	Right-side colon	Moderately	T4N0M0	*Wild type*	22.5
**PDX10**	31	F	Left-side colon	Poorly	T4N1M1	*Wild Type*	4.11
